# A PD-L1 Negative Advanced Gastric Cancer Patient With a Long Response to PD-1 Blockade After Failure of Systematic Treatment: A Case Report

**DOI:** 10.3389/fimmu.2021.759250

**Published:** 2021-12-07

**Authors:** Fangyuan Zhang, Jieying Zhang, Lei Zhao, Menglan Zhai, Tao Zhang, Dandan Yu

**Affiliations:** Cancer Center, Union Hospital, Tongji Medical College, Huazhong University of Science and Technology, Wuhan, China

**Keywords:** advanced gastric cancer, immunotherapy, PD-L1, lymph node metastasis, MSS, pMMR

## Abstract

**Background:**

It was widely accepted that programmed death-ligand 1 (PD-L1) positive, tumor mutational burden-high (TMB-H) or microsatellite instability-high (MSI-H) tumor are prone to have better treatment response to immune checkpoint blockade. The value of immune checkpoint blockade in PD-L1 negative gastric cancer patients has been questioned due to lower objective response rate (ORR).

**Case Presentation:**

We report an unusual case of a PD-L1 negative, proficient mismatch repair (pMMR)/microsatellite stability (MSS), tumor mutational burden-low (TMB-L) gastric cancer patient who achieved good response to immune checkpoint blockade after failure of systematic treatment. Multiple lymph nodes and bone metastases are the main characteristics of this patient. The patient survived for more than 30 months after diagnosis.

**Conclusions:**

This case suggested that PD-L1 negative gastric cancer patient may also benefit from immune checkpoint blockade. In gastric cancer, patients with lymph node metastasis may be potential beneficiaries.

## Case Presentation

A 39 years old man was admitted to our hospital on January 2, 2019 with the chief complaint of recurrent diarrhea and black stool for 3 weeks. A gastroscopy showed erosion, hyperplasia and protuberance of cardia mucosa. Pathological biopsy of the protuberant lesions showed cardia adenocarcinoma. Further immunohistochemistry showed MSH2 (+), MSH6 (+), PMS2 (+), MLH1 (+), HER-2 (2+). And negative for HER-2 fluorescence *in situ* hybridization (FISH) test and Epstein-Barr encoding region (EBER) polymerase chain reaction (PCR) test. A positron emission tomography - computed tomography (PET-CT) was conducted and showed thickened gastric cardia wall, multiple enlarged lymph nodes with high standard uptake value (SUV), including left supraclavicular lymph nodes, multiple lymph nodes adjacent to the cardia and retroperitoneal lymph nodes, multiple destructive bones (left scapula, right 5th rib, Th8-10 vertebrae, L3 left adnexa, sacrum and left iliac bone). He denied any family history of hereditary diseases and special medical history. The laboratory examination showed elevated carbohydrate antigen 199 (CA19-9) of 52.1U/ml and normal carcinoembryonic antigen (CEA), carbohydrate antigen 125 (CA125) and alpha fetoprotein (AFP) level.

**Figure 1:**
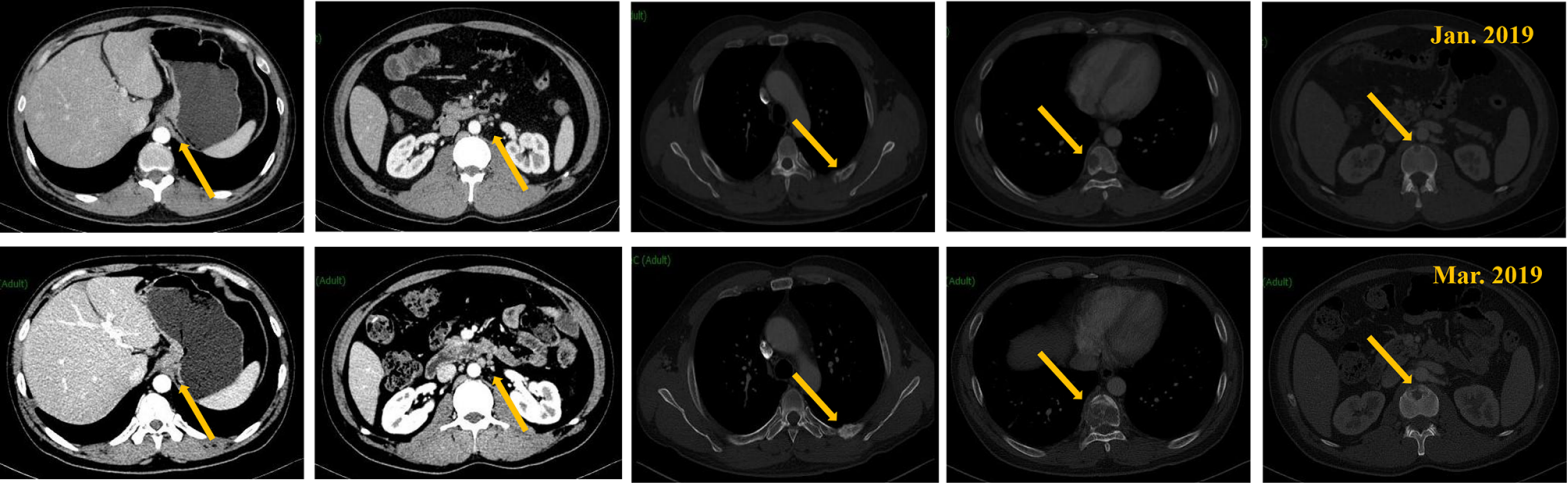
CT scan of the tumors before and after first-line treatment.

After admission, the patient received 4 cycles of FOLFOX4 chemotherapy from January 9, to February 27, 2019. After the initial 4 cycles of chemotherapy, the patient complaint of aggravated chest and back pain. Besides, CA125 and CA19-9 were increased from 32.1 U/ml to 62.9 U/ml and from 52.1 U/ml to 78.4 U/ml respectively. A computed tomography (CT) scan suggested an increase in the size of metastatic lymph nodes and the extent of bone metastases compared to that before treatment (**Figure 1**). Thus, the curative effect was evaluated as progressive disease (PD) with a progression-free survival (PFS) of 3.5 months.

**Figure 2:**
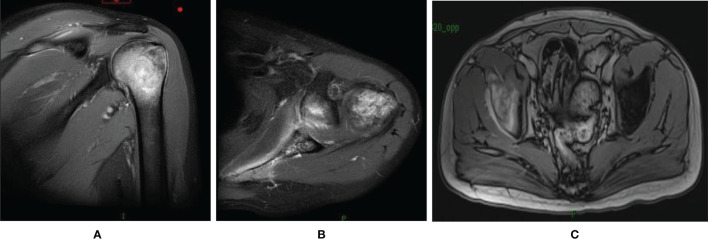
MRI of the shoulder joint and elvic showed newly formed bone metastases in the left humeral **(A, B)** and the right iliac **(C)**.

**Figure 3:**
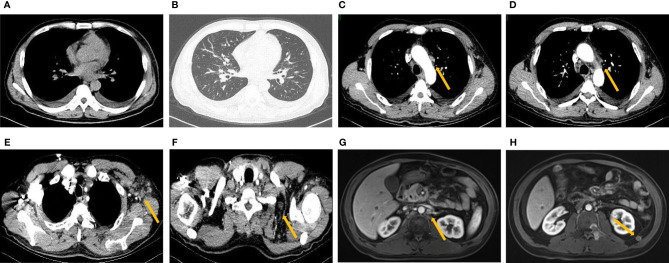
New pleural effusion **(A)** and cancerous lymphangitis **(B)** with enlarged mediastinal **(C, D)**, axillary **(E)** and supraclavicular **(F)** lymph nodes on pulmonary CT after third-line treatment. Enhanced MRI of the abdomen showed enlarged retroperitoneal lymph nodes **(G, H)**.

In order to look for more effective treatment, a next generation sequencing (NGS) (unpublished report) was performed and showed CCND1 gene amplification, RICTOR gene amplification, TMB-L (TMB 5 Muts/Mb) and MSS. The immunohistochemistry of PD-L1 with 22C3 antibody showed a Combined Positive Score (CPS) with 0 (**Supplementary Figure 1**). Then, the patient accepted second-line chemotherapy of albumin paclitaxel combined with tegafur (S-1) for 6 cycles from March 15^th^ to July 10^th^, 2019. In the meantime, thoracic spine and lumbar spine radiotherapy of DT 36Gy/12F was performed to relieve the pain. CT scans after every 2 cycles of chemotherapy suggested sustained stable disease (SD). CA125 and CA19-9 decreased to the normal level during the second-line chemotherapy gradually. The patient reported improving quality of life by relieving pain. Thus, single agent maintenance chemotherapy with S-1 was initiated from Aug. 2^nd^,2019. The regular visit 2 months later showed increased CA19-9 from 30.8 U/ml to 354 U/ml. And the pain was aggravated gradually. The CT scan suggested PD with newly formed bone metastases after maintenance chemotherapy with S-1 for only 2 months (**Figure 2**). Therefore, a third-line treatment was considered. And in the choice of third-line treatment regimen, either Apatinib or anti-PD-1 was considered according to the Chinese guideline at that time. While considering that the previous paclitaxel was effective as the second line therapy and the high cost of Apatinib or PD-1 antibody at that time, single-agent docetaxel was accepted as the final choice. Docetaxel was used for 3 cycles. And at the same time, right iliac and left acromioclavicular head radiotherapy was conducted at a dose of 30Gy/10F to relieve the pain. The evaluation after three cycles of third line docetaxel showed enlarged lymph nodes in the neck and axilla with aggravated cough, which suggested PD (**Figure 3**).

**Figure 4:**
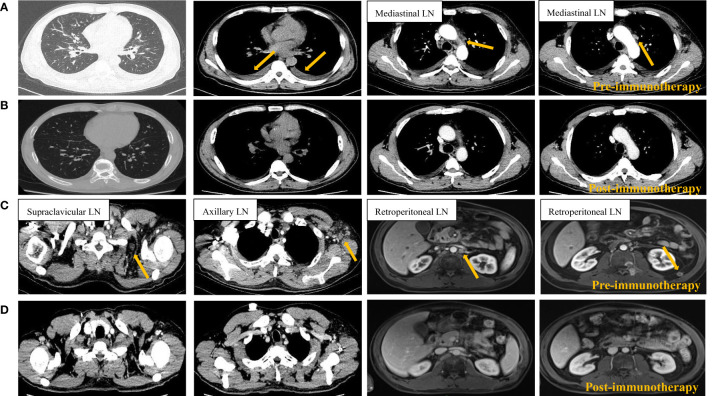
Radiological imaging of the tumors before **(A, C)** and after **(B, D)** 1 cycle of PD-1 blockade. Pre-immunotherapy tumor site indicated by orange arrow. LN, lymph node.

Luckily, a clinical trial of PD-1 antibody SCT-I10A (unlisted) for metastatic gastric cancer was recruiting in our center and the patient was a qualified participant. After informed consent, the patient took part in the clinical trial and received the first dose of PD-1 antibody (SCT-I10A) on January 10, 2020. Surprisingly, the patient reported that the cough disappeared around 3 weeks after the first dose of the drug. Then, due to the pandemic of Corona Virus Disease 2019 (COVID-19), the treatment was discontinued for nearly 3 months without any treatment. After the pandemic, the patient returned to the hospital for further evaluation. The result showed reduced pleural fluid, shrunken mediastinal, neck, axillary and retroperitoneal lymph nodes and normal tumor markers, which were suggestive of partial response (PR) (**Figure 4**). Considering the inspiring response, another 23 cycles of PD-1 antibody therapy were conducted with the last treatment on 2021.7.16 and the efficacy was assessed as sustained PR.

To further clarify whether this patient has specific gene mutations that make him sensitive to immunotherapy, a NGS test of peripheral blood was conducted on Jan. 28^th^, 2021. The results showed no gene mutations with MSS and TMB-L.

Up to now, the patient has an overall survival (OS) of over 30 months and has no discomfort. The timeline of patient treatment and change of tumor markers was shown in **Supplementary Figure 2**.

## Discussion

Worldwide, the incidence and mortality rate of gastric cancer are ranked fifth (5.6%) and fourth (7.7%) respectively among the most commonly diagnosed malignancies in 2020 (1). Immunotherapy aims at regulating the unbalanced anti-tumor immune response to restraint the growth of tumor and prolong the survival of patients. Several biomarkers and gene signature were proposed as predictors of better response to immunotherapy, such as high expression of PD-L1, mismatch repair deficiency (dMMR)/MSI-H, and high TMB. The result of KEYNOTE - 042 suggested patients with higher PD-L1 expression are associated with longer OS and higher objective response rate (ORR). In 2017, based on the result of 149 patients, Food and Drug Administration (FDA) approved pembrolizumab for MSI-H or dMMR solid tumor. Since then pembrolizumab became the first tissue/site agnostic immunotherapy. In the KEYNOTE - 158 study, 1073 patients with advanced incurable solid tumor who failed the standard therapy were enrolled to assess the association between tissue TMB and anti-tumor activity of PD-1 antibody pembrolizumab. This study prospectively proved that TMB-H group had higher ORR than the counterpart (29% vs. 6%) for the first time. Based on this study, FDA approved pembrolizumab for TMB-H solid tumor in Jun, 2020, which is the second tissue/site agnostic indication for immunotherapy. Although all the above evidence, there are always doubts about the predictive power of these factors. Because of the spatiotemporal heterogeneity of each tumor, the expression of PD-L1, status of TMB and MSI/MMR may not be a reliable predictor. The complexity of the choice of PD-L1 detection method and cut-off value of TMB further contributes to the inaccuracy of these predictors. Our case is just such an exception who is PD-L1 negative, pMMR/MSS and TMB-L, and achieved persistent good response to immunotherapy. It would be worthwhile to figure out the reason why the classic prognostic predictors failed in our case and reveal the underlying predictors.

In many types of tumors, PD-L1 expression was heterogenous between primary tumours and metastatic lymph nodes, and the expression rate of PD-L1 in the metastasis higher than that of the primary site (2–5). This discordance is actually exist in gastric cancer as well (6, 7). Unfortunately, we didn’t detect PD-L1 status in the lymph nodes of this patient.

Cancer chemotherapy drugs are often considered to be immune suppressive, but the reasonable time sequence of chemotherapy and immunotherapy may augment the therapeutic effect of immunotherapy (8–11). This may be due to the chemical exposure’s regulatory effect on T cell function (12–14). And Lukas W. Pfannenstiel deemed Paclitaxel can enhance immunity by promoting the maturation of dendritic cells (DC) cells (15). Previous studies have shown that Docetaxe-resistant tumor cells can increase susceptibility to cytotoxic T lymphocyte (CTL) killing through “ immunogenic modulation ” after treatment with docetaxel (16). These mechanisms may contributed to the patient’s sensitivity to immunotherapy, but it still need to be confirmed by a sophisticated designed clinical experiments.

There are also studies shown that RICTOR gene expression is associated with lymph node metastasis in gastric cancer (17, 18), which to some extent explains the prominent characteristics of lymph node metastasis of this patient. However, there is no evidence that RICTOR expression is associated with the efficacy of immunotherapy.

## Conclusion

We report a PD-L1 negative, pMMR and TMB-L gastric cancer patient who was highly sensitive to immunotherapy and had a long response, which is against the traditional opinion about the predictive factors of immunotherapy. For our case, the reasons may be as follows, (1) PD-L1 expression was heterogenous between primary tumours, or between primary cancer and metastatic lymph nodes. (2) Potential synergistic effects of previous chemotherapy on immunotherapy. (3) Higher responsiveness of lymph node metastases to immunotherapy. This case enlightens us to have a more comprehensive understanding of the prediction of immunotherapy.

## Data Availability Statement

The original contributions presented in the study are included in the article/**Supplementary Material**. Further inquiries can be directed to the corresponding author.

## Ethics Statement

Written informed consent was obtained from the individual(s) for the publication of any potentially identifiable images or data included in this article.

## Author Contributions

FZ and JZ were mainly responsible for the article writing. LZ, MZ, and TZ were responsible for patient’s clinical data and analysis. DY was the corresponding author. All authors contributed to the article and approved the submitted version.

## Funding

This study was supported by the National Natural Science Foundation of China (No. 81872429).

## Conflict of Interest

The authors declare that the research was conducted in the absence of any commercial or financial relationships that could be construed as a potential conflict of interest.

## Publisher’s Note

All claims expressed in this article are solely those of the authors and do not necessarily represent those of their affiliated organizations, or those of the publisher, the editors and the reviewers. Any product that may be evaluated in this article, or claim that may be made by its manufacturer, is not guaranteed or endorsed by the publisher.
