# Supplementary material for: A PD-L1 Negative Advanced Gastric Cancer Patient With a Long Response to PD-1 Blockade After Failure of Systematic Treatment: A Case Report
Source: Front Immunol. 2021 Dec 7;12:759250. doi: 10.3389/fimmu.2021.759250 (PMC8688253; doi:10.3389/fimmu.2021.759250)
Supplement: Supplementary file 1 [file Image_1.pdf]

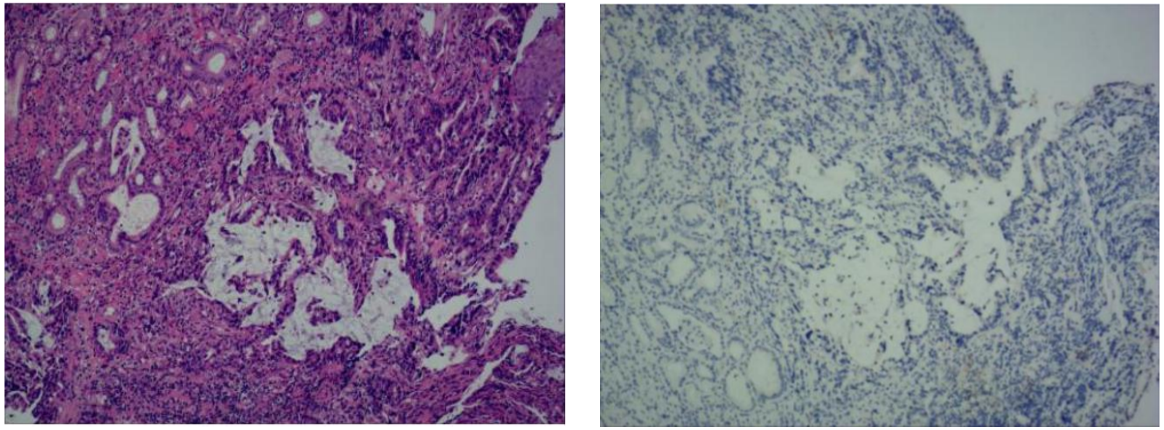

Supplementary Figure 1: Primary cancer of HE staining and PD-L1 testing.

The tumor cells were negative for PD-L1. PD-L1 CPS=0.

The PD-L1 expression was detected through a prototype IHC assay by using the 223C antibody.

CPS: PD-L1 stained cells (tumour cells, lymphocytes, macrophages) counts divided by all tumour cell counts multiplied by 100.
