# Supplementary material for: A PD-L1 Negative Advanced Gastric Cancer Patient With a Long Response to PD-1 Blockade After Failure of Systematic Treatment: A Case Report
Source: Front Immunol. 2021 Dec 7;12:759250. doi: 10.3389/fimmu.2021.759250 (PMC8688253; doi:10.3389/fimmu.2021.759250)
Supplement: Supplementary file 2 [file Image_2.pdf]

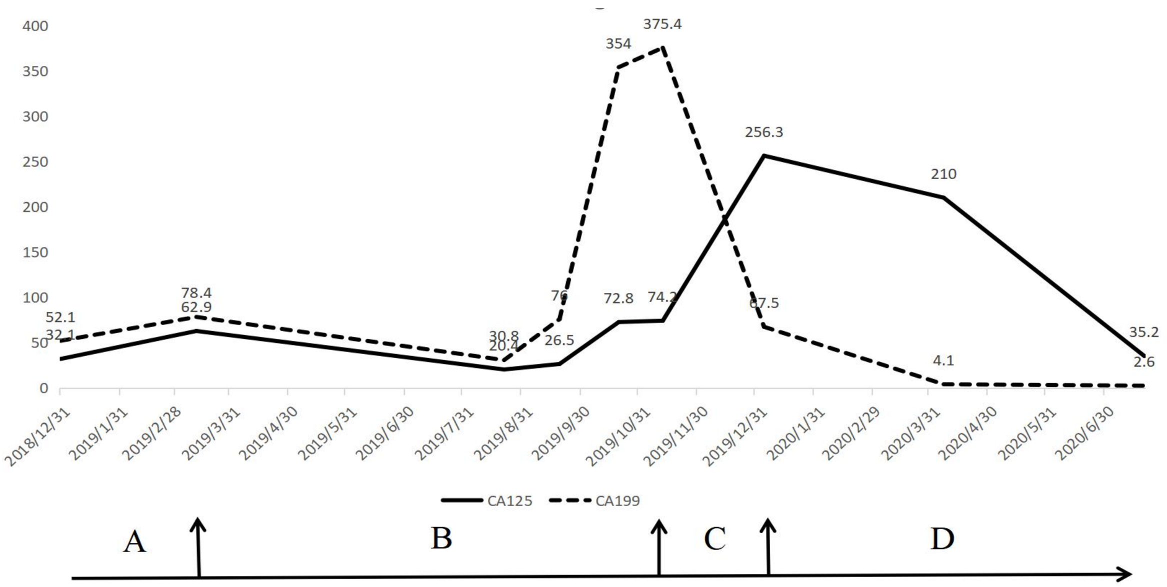

Supplementary Figure 2: Timeline of patient treatment and change of tumor markers.

A: First-line treatment (FOLFOX4), PFS: 3.5 months.

B: Second-line treatment (Albumin paclitaxel combined with tegafur + maintenance chemotherapy with tegafur), PFS: 7 months.

C: Third-line treatment (Docetaxel), PFS: 2.5months

D: Fourth-line treatment (PD-1 antibody).
